# Remodelling of the healthy foal’s conjunctival microbiome in the first two months of life

**DOI:** 10.2478/jvetres-2025-0001

**Published:** 2025-01-31

**Authors:** Katarzyna Płoneczka-Janeczko, Eve Armstrong, Marta Siemieniuch-Tartanus, Marcin Magdziarz

**Affiliations:** Department of Epizootiology with Clinic of Birds and Exotic Animals, Wrocław University of Environmental and Life Sciences, 50-366 Wrocław, Poland; Faculty of Veterinary Medicine, Wroclaw University of Life Science, 50-375 Wrocław, Poland; Department of Large Animal Diseases and Clinic, Institute of Veterinary Medicine, Warsaw University of Life Sciences, 02-797 Warsaw, Poland; Hugo Steinhaus Center, Faculty of Pure and Applied Mathematics, Wrocław University of Science and Technology, 50-376 Wrocław, Poland

**Keywords:** microbiota, ocular surface, horses, foals, NGS

## Abstract

**Introduction:**

The aim of the study was to explore and characterise healthy foals’ eye microbiomes in the first two months of life.

**Material and Methods:**

Conjunctival swabs were collected three times, not later than 12 h after delivery and again at the end of the first and the second months of life from six clinically healthy foals of the Polish Konik breed. The average interval between the first and second samplings was 33.3 days and between the second and third was 35.6 days. Next-generation sequencing performed on a MiSeq sequencer in paired-end technology was used to analyse the composition of the conjunctival microbiota.

**Results:**

Paired one-sided *t*-tests revealed that conjunctival microbiota diversity was the lowest in the first 24 h of life and significantly increased between birth and the first month. The most prevalent family throughout the study was *Micrococcaceae* and the most prevalent genus was *Corynebacterium*. Sequences of potentially pathogenic bacteria such as *Pseudomonas, Acinetobacter* and *Streptococcus* spp. that may be involved in inflammatory processes were identified. Ocular commensals such as *Corynebacterium* and *Lactobacillaceae* that were found in the ocular surface microbiome of the foals are believed to be capable of restoring the ocular microbiome and maintaining balance.

**Conclusion:**

A healthy ocular surface microbiota in the early period of a foal’s life develops dynamically and changes its composition.

## Introduction

Normal foals are born with the eyelids open (6), and this state makes checking the eye condition of newborn foals possible; this is an important part of their first veterinary examination. Based on examination using the Schirmer test, foals within the first 48 h of birth have lower lacrimal secretion than their mothers, and this does not appear to increase during the first month of life (18). The conjunctiva of foals is very vulnerable to colonisation or contamination by various microorganisms, including those from the vaginal environment of their mares and the skin of the genital (labia) and the perianal areas, as well as those from the oral cavity (which are transferred by the mare licking the foal after birth) or the soil. The microbiome of horses, particularly that of the large intestine, has been intensively studied in the last decades, and the positive effect of stability in the microbiome not only on the digestion of feed, but also on the health of the horse is widely accepted (2). The ocular surface microbiota (OSM) is fundamentally important for eye homeostasis and health. The most common foal conjunctival diseases recognised in the juvenile period include conjunctivitis, subconjunctival haemorrhages and inflammations. Similarly to how they protect other mucous membranes, nonpathogenic commensal conjunctival microorganisms protect the eye against pathogens by producing antibacterial substances or by competitive niche occupation, reducing access to nutrients (36). Prevention of the proliferation of pathogenic species correlates with maintenance of the microbiota’s composition, and it is suggested that any alterations or changes here may predispose the eye to the development of several diseases (23). Little evidence has been reported on the composition of the ocular microbiota of term newborn foals born under field conditions. Before high-throughput techniques were introduced for the sequencing of the 16S rRNA gene, the ocular surface was evaluated using traditional culture-based methods. Studies carried out on infants’ ocular microbiota have demonstrated that at birth, the conjunctival microbiota is very similar to that of the uterine cervix, but that two days after, the ocular microbiota begins to change and evolve throughout childhood, finally taking on a composition close to that of adults’ microbiota (23). Therefore, changes do not occur as quickly after delivery as it might seem, despite the permanent contact of the newborn with its environment and mother. Analysis of the composition of the juvenile conjunctival microbiome is essential for better understanding of its potential roles in the protection from or development of several eye diseases, also in foals. Knowledge of this topic can contribute to the development of new standards in newborn foals’ ocular prophylaxis and prevention of neonatal eye diseases. In this context, the aim of our research was to explore and characterise the OSM community structure in newborn foals during the early stages of their life (from birth to the end of the second month). This updates and expands the knowledge of foal ophthalmology.

## Material and Methods

Research was carried out under field conditions on six foals of the Polish Konik breed born at the stud farm owned by the Research Station of the Institute of Animal Reproduction and Food Research of the Polish Academy of Sciences in Popielno, Poland. The births took place at night, without complications for any offspring or dams and without any assistance or care from humans. All foals were born clinically healthy, and their dams did not show any signs of disease during pregnancy or after birth.

To estimate the structure and changes in the eye microbiome of newborn foals, a clinical investigation was conducted. The procedures performed in the project did not need the approval of the Institutional Animal Care and Use Committee, which was confirmed by the Local Ethical Committee at the University of Warmia and Mazury in Olsztyn with edict LKE.31.01.2020. Samples were taken longitudinally, not later than 12 h after delivery (time point I) and again after the first (time point II) and second (time point III) months of life (Supplementary Table 1). Samples from the left and right eye were individually collected from the ventral conjunctival fornix using sterile cotton swabs soaked in sterile 0.9% saline (Sarstedt, Copan, Brescia, Italy) (34). Special care was taken not to contaminate the swabs by contact with the eyelids or eyelashes. The left and right eye of each foal was swabbed once and the procedure was duplicated. A total of 24 swabs (4 per animal) were collected during each sampling. Specimens were quickly placed in cryogenic tubes at a temperature of −20°C for freezing and storage. After the conjunctival swab collection was completed, samples were shipped in an expanded polystyrene box with a cooler (at a temperature of 2–4°C) by courier directly for analysis (Genomed, Warsaw, Poland). The swabs were not frozen again. The same method of swabbing and storage is commonly used in human medicine to avoid contamination with other microbiota (3).

Genomic DNA was isolated immediately after delivery of samples using a Genomic Mini AX Bacteria kit (A&A Biotechnology, Gdańsk, Poland) according to the manufacturer’s instructions, with an additional mechanical lysis of each sample effected by zircon balls in a FastPrep homogeniser (MP Biomedicals, Irvine, CA, USA), using a previously reported procedure (28). Concentration of DNA was measured through the fluorometric method, using a Qubit 4 fluorometer (Thermo Fisher Scientific, Carlsbad, CA, USA). The presence of bacterial DNA was confirmed in a qPCR reaction, using 1055F (5′-ATGGCTGTCGTCAGCT-3′) and 1392R (5′-ACGGGCGGTGTGTAC-3′) universal primers for 16S rRNA (5) and demineralised water as a negative control. The metagenomic analysis of bacteria and Archaea was based on the amplification of the V3–V4 hypervariable region (encompassing approximately 469 base pairs) of the 16S rRNA gene. For the amplification of the selected region and the preparation of DNA libraries, a pair of 341F and 785R primers and NEBNext Q5 Hot Start High-Fidelity DNA Polymerase (New England Biolabs, Ipswich, MA, USA) were used. For the measurement of DNA concentration, 1μL of the reaction mixture was taken immediately after the PCR. The unpurified product contained the reaction mixture with primers and primer dimers; therefore, the result for the negative control was not zero. Concentrations up to a value of approximately 1.5 ng/μL were considered acceptable. When the negative control was above this value (indicative of contamination with PCR reagents) the reaction was repeated. Arbitrarily, it was assumed that the lowest DNA concentration for the testing sample should be twice the minimal concentration of the negative control. Finally, purification of samples was performed with AMPure XP (Beckman Coulter, Indianapolis, IN, USA), followed by final DNA measurement by the fluorometric method on a Tecan reader (Männedorf, Switzerland). A PCR was carried out to index DNA in 50 μL reaction volumes. Next-generation sequencing (NGS) was performed on a MiSeq sequencer (Illumina, San Diego, CA, USA) in paired-end technology (PE300) by Genomed (Warsaw, Poland). MiSeq Reporter v.2.6 software (Illumina) was used for data analysis. To ensure the classification of reads at the species level, bioinformatic analysis was carried out with QIIME software (1), a semiquantitative approach to microbial ecology based on the SILVA v.138 database of reference sequences (24). Data analysis was performed of all sequences obtained from NGS and filtered. The diversity of the foals’ microbiota was analysed using alpha (Shannon and Simpson) and beta (Bray–Curtis) diversity indices (9). The diversity of microbial communities was compared in two ways: in all individuals between samplings and as a mean for the group between samplings, using sequence reads. All diversity indices were calculated for all six foals. Paired one-sided *t*-tests were used to verify statistically significant changes in biodiversity between collected samples. The calculations were made separately at the family, genus and species taxonomic levels. All calculations were performed in the MATLAB R2020a environment (MathLabs, Natick, MA, USA). Taxa present in amounts equal to or greater than 1% of the total identified DNA sequences at least in one of the individuals were classified as abundant, as proposed by Kim *et al*. (11). If the proportion of identified sequences was <1%, taxa were classified as nonabundant.

## Results

### DNA sequence data

A total of 2,203,951 qualitatively evaluated 16S rDNA sequences were obtained for all samples collected from foals. Sequences of DNA were analysed for each individual foal at three time points. Supplementary Table 2 presents detailed information on the raw-read pairs, reads that passed final quality checks, and number of operational taxonomic units (OTUs) per sample for each animal at time points I, II and III. The mean number of OTUs per sample was 97,563, and the range was from 70,140 to 135,629.

### Microbial community composition

Data from all conjunctival samples were analysed both overall as a group and individually. Classification rate by taxonomic level (at least 99% sequence similarity) showed that over examined time, the mean percentage of the total identified sequences increased from 73.00% to 77.35%. During subsequent samplings, it was noticed that there was a higher prevalence of identified sequences at the kingdom taxonomic level than at the species level, where there was a decreasing trend over time and lower percentage of identified sequences. The proportions of bacteria were almost identical during the analysed life period of the foals (99.55% at time point I, 99.77% at time point II and 99.77% at time point III). Archaea were generally minimally present and were classified as non-abundant. Non-assigned sequences constituted a small percentage (<1%). Several differences in the abundance of bacterial taxa were detected between individual animals at the same time points as well as between successive samplings. Sequences representative of 12 bacterial phyla were identified, with individual foals having a total of representative sequences in the range of 7 to 11. The numbers of determined phyla differed least at the end of the first month of the foals’ lives. In all animals, regardless of age, *Actinobacteriota* (39.62% at time point I, 31.53% at time point II and 29.30% at time point III), *Firmicutes* (26.34%, 26.47% and 22.28%), *Proteobacteria* (16.39%, 21.09% and 32.39%), *Bacteroidota* (7.04%, 9.07% and 6.32%) and *Verrucomicrobiota* were always detected as the most abundant phyla.

**Fig. 1.**
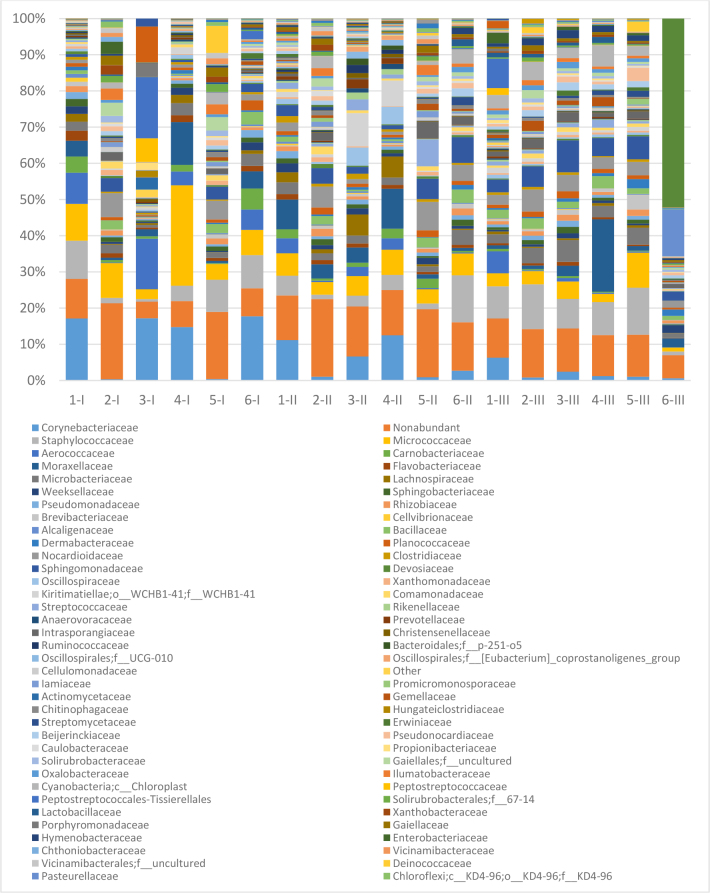
Prevalent bacterial families in foals’ conjunctival microbiota identified in individual animals. 1–6 – individual foals; I – first sampling time point (within 12 h of birth); II – second sampling time point (after the first month of life); III – third sampling time point (after the second month of life)

The examined foals’ conjunctiva were found to have a total of 75 prevalent bacterial families, with a range of 12 to 33 families for each individual foal. Figure 1 presents prevalent families ordered row-wise from highest to lowest bacterial abundance (according to how they presented in the time-point I sample from foal 1). When at least one of the foals showed an abundance of one family taxon during the same sampling, a trend of reduction in diversity was observed (53, 47 and 46 families). The most prevalent families (with a mean for the group >1%) throughout the study were *Micrococcaceae, Staphylococcaceae, Corynebacteriaceae, Moraxellaceae, Sphingomonadaceae* and *Nocardioidaceae*.

The abundance ranking in the first 24 h of life was similar and led by *Corynebacteriaceae* at 11.27%, after which came *Micrococcaceae* (10.26%), *Staphylococcaceae* (5.86%), *Aerococcaceae* (5.36%) and *Moraxellaceae* (4.03%). At the end of the foals’ second month, three of these families were still high in abundance, but two different families were also in notable presence. Their order at that time was *Staphylococcaceae* (8.77%), *Cardiobacteriaceae* (8.69%), *Sphingomonadaceae* (5.31%), *Moraxellaceae* (4.76%) and *Micrococcaceae* (4.18%). Variation between individuals in relation to age at the level of family was also observed.

**Fig. 2.**
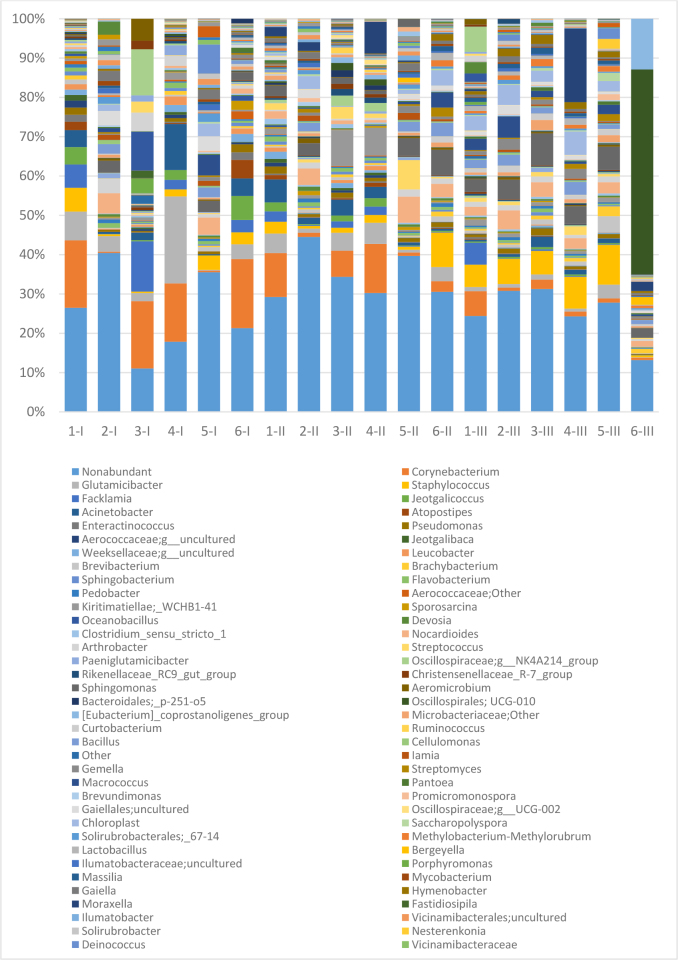
Prevalent bacterial genera in foals’ conjunctival microbiota identified in individual animals. 1–6 – individual foals; I – first sampling time point (within 12 h of birth); II – second sampling time point (after the first month of life); III – third sampling time point (after the second month of life)

At the genus level, a total of 89 prevalent genera were identified, with between 60 and 85 having been noted in individual foals. Differences between subsequent samplings were also detected. When in at least one of the foals during the same sampling a genus taxon could be identified as abundant (>1%), 55, 56 and 42 genera were present at time points I, II and III, respectively (Fig. 2). The most prevalent genera (with a mean for the group >1%) identified throughout the study were *Corynebacterium, Glutamicibacter, Nocardioides, Bacillus, Facklamia, Staphylococcus, Sphingomonas* and *Acinetobacter*.

The four genera in heaviest presence in the first 24 h were also among these eight and were *Corynebacterium* (11.23%), *Glutamicibacter* (6.59%), *Facklamia* (4.09%) and *Acinetobacter* (3.87%).

### Microbial diversity

**Fig. 3.**
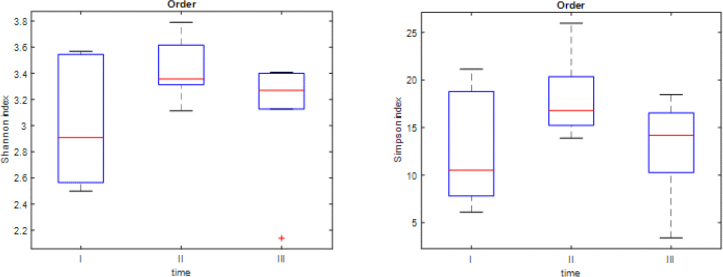
Box plots presenting Shannon and Simpson indices at the order level. The red line represents the median for all six foals

**Fig. 4.**
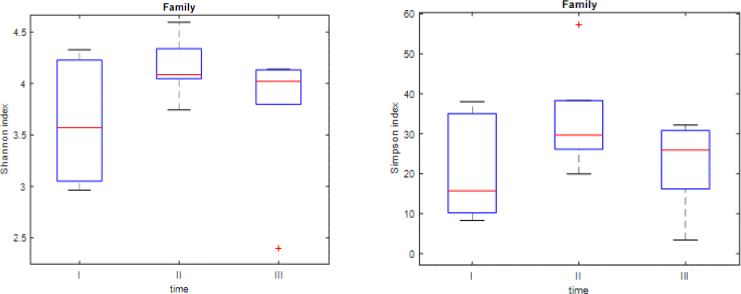
Box plots presenting Shannon and Simpson indices at the family level. The red line represents the median for all six foals

**Fig. 5.**
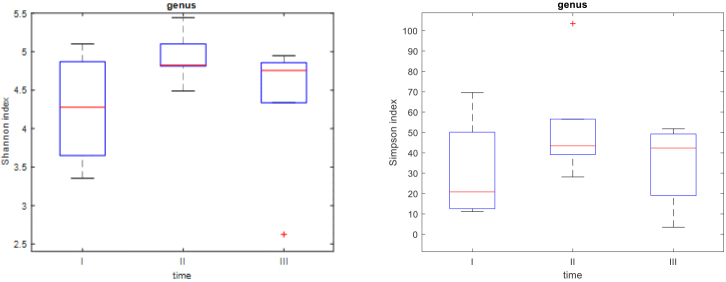
Box plots presenting Shannon and Simpson indices at the genus level. The red line represents the median for all six foals

The Shannon and Simpson indices demonstrated changes in the biodiversity at the order (Fig. 3), family (Fig. 4) and genus (Fig. 5) taxonomic levels. The obtained results proved that the biodiversity at time point II was significantly higher than at time point I. The Simpson indices also indicated that the biodiversity at time point II was significantly higher than at time point III. There was no significant difference in biodiversity between the swab samples from time points I and III (Tables 1–3). At the beginning of the study (time point I), there was high between-foal variation in biodiversity levels, while in subsequent samplings (time points II and III) the between-foal variation in biodiversity became lower.

**Table 1.** P-values of the *t*-tests applied to compare Shannon and Simpson indices at different time points at the order taxonomic level

	I *vs*. II	I *vs*. III	II *vs*. III
Shannon index	0.0048*	0.3616	0.0863
Simpson index	0.0058*	0.4607	0.0369*

1 * – <0.05 α significance level; I – first sampling time point (within 12 h of birth); II – second sampling time point (after the first month of life); III – third sampling time point (after the second month of life)

**Table 2.** P-values of the *t*-tests applied to compare Shannon and Simpson indices at different time points at the family taxonomic level

	I *vs*. II	I *vs*. III	II *vs*. III
Shannon index	0.0074*	0.3632	0.0998
Simpson index	0.0024*	0.3866	0.0459*

1 * – <0.05 α significance level; I – first sampling time point (within 12 h of birth); II – second sampling time point (after the first month of life); III – third sampling time point (after the second month of life)

**Table. 3.** P-values of the *t*-tests applied to compare Shannon and Simpson indices at different time points at the genus taxonomic level

	I *vs*. II	I *vs*. III	II *vs*. III
Shannon index	0.0073*	0.3968	0.0907
Simpson index	0.0016*	0.3557	0.0703

1 * – < 0.05 α significance level; I – first sampling time point (within 12 h of birth); II – second sampling time point (after the first month of life); III – third sampling time point (after the second month of life)

These results show that the biodiversity at time point II was significantly higher than the biodiversity at I. The Simpson index also indicated that the biodiversity at time point II was significantly higher than the diversity at III. There was no significant difference in biodiversity between samples taken at time point I and those taken at time point III.

These results show the same pattern of biodiversity and time points of sampling as observed at the order taxonomic level.

These results once again show that the biodiversity at time point II was significantly higher than the biodiversity at I and that there was no significant difference in biodiversity between time-point-I samples and time-point-III samples. The P-values of the tests comparing time points II and III are very close to the significance level of 0.05, and thus concur with the evidence in Fig. 5 that the biodiversity at time point III decreased from its level at time point II.

**Fig. 6.**
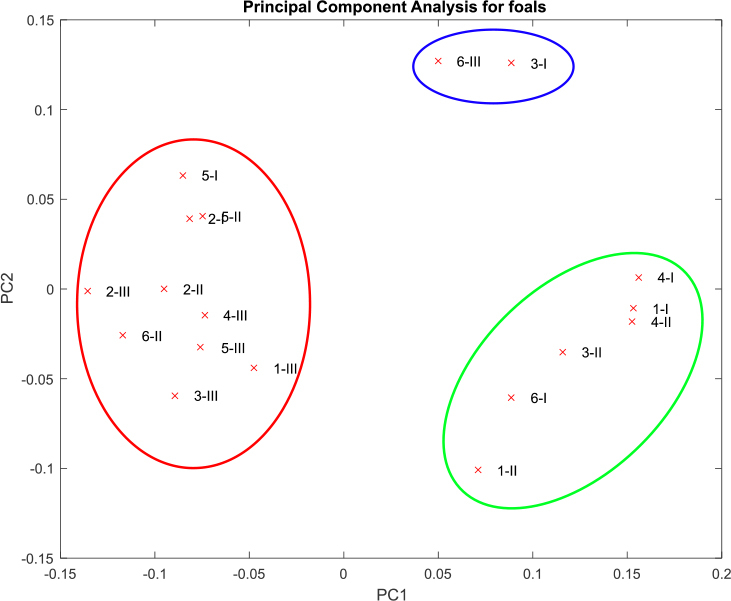
Principal component analysis of the Bray–Curtis distance table on the species level

The Bray–Curtis distance (weighted) answers whether shared taxa have similar abundances. Evaluating compositional abundance with this distance showed that the samples from foal 3 at time point I and foal 6 at time point III differed markedly from the rest (Supplementary Table 3, Fig. 6). It should be noted that the conjunctival microbiomes of foals formed three independent clusters, regardless of the sampling time (Fig. 6).

## Discussion

Currently, knowledge regarding the microbiome of any newborn foal tissue is limited. This is the first report that describes the OSM in the first 24 h of a foal’s life, and it provides a new *status quo* in veterinary neonatal ophthalmology. Conclusions drawn from microbiome studies should, however, be treated with caution because the experimental methods which led to them have not been fully standardised. Variability of procedure at each step in the execution of microbiome research, from the collection and storage of samples to DNA extraction, PCR library construction, sequencing, bioinformatics and statistical analyses, introduces variability and biases in the results. Ultimately, this lack of standardisation makes inter-study comparison extremely challenging.

The ocular surface of horses was often described as poorly colonised, and no growth of bacteria was obtained from samples collected from healthy eyes when traditional culture methods were applied (7, 16, 29). The advent of molecular techniques based on 16S rRNA sequencing opened up the possibility of determining the wide spectra of bacteria inhabiting the conjunctiva of humans (4) and healthy horses (12). The present study confirmed that almost immediately after birth, the ocular surface of healthy foals contained a greater diversity of microbiota than had been identified in culture-based methods (36). Our research identified uncultured genera belonging to the *Vicinamibacteraceae, Ilumatobacteraceae, Weeksellaceae, Aerococcaceae, Peptostreptococcaceae, Xanthobacteraceae* and *Cardiobacteriaceae* families, which have been identified not only in tissues and products of animal origin, but also in very diverse environments, *e.g*. soil and water (8, 13, 14, 17, 20, 21, 22, 26, 31, 32). Many of their functions have not yet been discovered and remain unknown.

Some microorganisms represent persistent conjunctival commensals or pathogens, but others may only constitute a transient microflora (25). Most studies focused on the conjunctival microbiota (12, 27) or mycobiota (35) of adult healthy horses. To the best of our knowledge, little evidence has been reported on the composition of the ocular microbiota of newborn foals, which makes it virtually impossible to compare our research results with those of others. The only studies to date involving the ocular surface microbiota of foals (ranging 2–6 months in age) were based on culture techniques (36). The number of bacterial phyla identified in our study was much lower than was identified in adult horses (12 *vs*.17) (27), which can be explained by age- and maturity-related changes of the microbiome composition. Also, the most abundant phylum in adults is different to that in foals: *Pseudomonadota* were the most numerous in adult horses (27), while in the foals in our study, the most numerous group was *Actinobacteriota*. Similarly the percentage of *Proteobacteria* in foals in our study was lower (15%) than that in adult horses (46.1%) (29). These differences suggest that the neonatal ocular microbiota could undergo changes over time and that the early-life period is a prime time for the colonisation of the conjunctiva surface in foals.

The ability to identify a set of core microbial taxa in healthy individuals is very important from the perspective of clinical ophthalmology. According to the definition in the literature, the “core microbiome” refers to any set of microbial taxa, or the genomic and functional attributes associated with those taxa, that are characteristic of a host or environment of interest. To allow comparison of core taxonomic host microbiomes, the same criteria should be applied for quantifying the cores in all instances for comparison (19). Our evidence showed that although the family prevalence ranking of *Corynebacteriaceae, Micrococcaceae, Staphylococcaceae, Aerococcaceae* and *Moraxellaceae* in the first 24 h of life had not changed after the first month of life and only percentage decreases were noted, it had changed after the second month of life and was *Staphylococcaceae, Cardiobacteriaceae, Sphingomonadaceae, Moraxellaceae* and *Micrococcaceae* at that time. Therefore, it is very clear that the OSM of foals rather represents a “dynamic” than a “core” microbiome, that, in view of its prevalent taxa, was being radically transformed. Different results were shown for prevalent bacteria in adult equine OSM by Scott *et al*. (29): the most abundant bacterial families identified on the eyeball surface were *Pasteurellaceae* (13.7%), *Sphingomonadaceae* (7.9%) and unclassified members of the *Cardiobacteriales* order (7.7%). Other families present in 96–100% of the eyes included unclassified members of the *Bacteroidales* order (5%), *Moraxellaceae* (4.8%), *Ruminococcaceae* (4.5%) and *Gamellaceae* (4.1%) (29); with the exception of *Moraxellaceae* (which was at comparable abundance with our 4.76%), these families were all low abundant in newborn foals.

In order to help identify the core microbiome, the taxa which are shared across samples varying temporally may be defined, as proposed by Shade and Handelsman (30); however, these constituents may only be discernible at a higher taxonomic level. In regard to this identification scheme, the family detected in our study in the highest proportion (100%) of all eyes examined at the three sampling time points was *Micrococcaceae*. The lack of coincidence of the prevalent core families in adult and in very young animals suggests that the existence of separate microbiome frameworks related to age should be considered. On the other hand, our study discovered three clusters of microbiota species which were always together irrespective of sampling time, which was surprising and unexplainable for the present.

To the best of our knowledge, the limitation of microbiome research based on the 16S subunit is that results involve margins of error when analysis extends below genus level and attempts to define species. Therefore it is difficult to say with certainty whether the bacteria identified in our studies were clearly pathogenic or nonpathogenic. Ocular commensals such as *Corynebacterium* spp., coagulase-negative *Staphylococcus* spp. and *Lactobacillaceae* are typically claimed to be capable of restoring the ocular microbiome and maintaining balance (15). *Corynebacterium* mitigates the pathological effects of pathogenic bacteria within the conjunctiva. However, *Corynebacterium* spp. have the potential to cause serious eye infection in some conditions (33). In our study *Lactobacillus* was present in all individuals but non-abundant in the first sampling, present only in 83% of animals at the second time point, but more extensively present at the third sampling as the ocular surface microbiome matured. An important finding of this study was that through the analysis, a decreasing tendency in subsequent samplings (means of 11.23%, 5.82% and 2.07%) was observed for *Corynebacterium*, which may affect the homeostasis between commensal microorganisms. We also found an upward trend for *Staphylococcus*, with its mean proportion increasing from 2.53% to 6.12% and its instance rising from in 66% to in 83% of the examined eyes as the microbiome matured. Sequences for less beneficial bacteria such as *Pseudomonas* spp., *Acinetobacter* spp. and *Streptococcus* spp. that may be involved in the inflammatory processes were also identified. The first of these was present in all samplings at a similar level of approximately 1%. *Pseudomonas* spp. may be constituents of the normal flora of mucous membranes, skin and intestinal contents. However, *Pseudomonas aeruginosa* may be a second cause, after *Streptococcus equi*, of ocular ulcerative keratitis in horses (10). The composition of the early-life eye microbiota is probably shaped by many factors. It should be said that a limitation of the present study is the lack of microbial analyses of the bedding material in the boxes, with which the newborn foal’s eye may come into contact. Similarly, the bacterial oral cavity microbiomes of the dams were not investigated, as it was not the main goal of our study. The lack of subtractive filtering to remove contaminants, even when negative controls failed to show amplification in PCR, may also have allowed potential reagent and environmental contaminants to confound the interpretation of microbiome data. For this reason, the questions of when the microbiota colonises the mucous membranes of the newborn foal and where the bacteria come from arise. Although a relatively small number of individuals were taken into account, the research group consisted of foals born at a similar time, of the same sex, and from mothers kept in the same conditions. Therefore, we assume that this was a fairly homogeneous group.

## Conclusion

Despite the potential of NGS analysis, the conjunctival microbiota of foals still needs more particular species-specific characterisation with the higher accuracy of whole-genome sequencing (WGS). The accumulation of more data as proposed in this study is also needed in order to better understand eye homeostasis and early development of pathologies.

## Supplementary Material

Supplementary Material Details
