# Supplementary material for: Remodelling of the healthy foal’s conjunctival microbiome in the first two months of life
Source: J Vet Res. 2025 Jan 31;69(1):131–40. doi: 10.2478/jvetres-2025-0001 (PMC11936096; doi:10.2478/jvetres-2025-0001)
Supplement: Supplementary file 1 — Supplementary Material Details [file jvetres-2025-0001_sm.pdf]

Supplementary Table 1. Characteristics of examined foals and time of sampling

Foal	Mare age (years)	Foal sex	Foal weight on the 3rd day after delivery	Date of sampling		
				I	II	III
1	10	female	35 kg	12/03/2020	16/04/2020	25/05/2020
2	16	female	44.3 kg	14/03/2020	16/04/2020	25/05/2020
3	7	female	34 kg	16/03/2020	16/04/2020	25/05/2020
4	5	female	38 kg	16/03/2020	16/04/2020	25/05/2020
5	11	female	35 kg	19/03/2020	21/04/2020	20/05/2020
6	9	female	35 kg	15/04/2020	21/05/2020	20/06/2020

I – first sampling time point (within 12 h of birth); II – second sampling time point (after the first month of life); III – third sampling time point (after the second month of life)

Supplementary Table 2. Summary of sequencing data for each foal in the first 24 h of life (I) and at the end of the first (II) and second (III) months of life

Foal	Raw-read pairs	Passing QC	OTU/sample OTU/qualitative	Raw-read pairs	Passing QC	OTU/sample OTU/qualitative	Raw-read pairs	Passing QC	OTU/sample OTU/qualitative
1	119,688	119,031	94,984/4,453	113,545	112,569	89,365/5,806	116,766	116,171	93,189/6,081
2	135,843	13,184	84,343/6,807	138,933	138,320	102,202/8,261	88,526	88,075	70,140/5,449
3	171,119	170,161	135,629/3,788	140,410	139,668	109,042/6,826	141,822	141,080	110,466/6,709
4	165,392	164,595	104,829/4,972	145,387	144,651	112,129/6,497	90,879	90,436	72,743/4,311
5	119,510	118,807	89,156/5,810	135,611	134,882	99,478/6,598	149,018	148,310	98,167/7,082
6	135,225	134,409	107,295/5,789	97,416	96,853	75,565/4,877	133,419	132,749	107,427/3,893

OTU – operational taxonomic units; Passing QC – percentage of reads remaining after QC filtering using fastp to remove low quality bases, short reads (<35 base pairs) and low-complexity reads

Supplementary Table 3. Bray–Curtis distances showing compositional heterogeneity of bacterial communities among all foals at the species level; the higher the Bray–Curtis index, the bigger the difference between microbiomes

	1-I	1-II	1-III	2-I	2-II	2-III	3-I	3-II	3-III	4-I	4-II	4-III	5-I	5-II	5-III	6-I	6-II	6-III
1-I	0.00	0.41	0.64	0.81	0.74	0.76	0.72	0.51	0.65	0.38	0.42	0.75	0.84	0.81	0.68	0.37	0.72	0.92
1-II	0.41	0.00	0.54	0.64	0.57	0.58	0.74	0.35	0.52	0.44	0.40	0.60	0.70	0.64	0.56	0.36	0.57	0.78
1-III	0.64	0.54	0.00	0.59	0.57	0.42	0.66	0.64	0.46	0.72	0.68	0.46	0.60	0.62	0.49	0.59	0.41	0.76
2-I	0.81	0.64	0.59	0.00	0.36	0.45	0.89	0.73	0.52	0.79	0.78	0.61	0.36	0.42	0.58	0.68	0.48	0.76
2-II	0.74	0.57	0.57	0.36	0.00	0.44	0.85	0.66	0.45	0.75	0.70	0.52	0.37	0.35	0.52	0.63	0.49	0.74
2-III	0.76	0.58	0.42	0.45	0.44	0.00	0.88	0.69	0.37	0.81	0.77	0.37	0.41	0.47	0.42	0.64	0.30	0.72
3-I	0.72	0.74	0.66	0.89	0.85	0.88	0.00	0.75	0.82	0.75	0.75	0.88	0.93	0.89	0.85	0.76	0.86	0.95
3-II	0.51	0.35	0.64	0.73	0.66	0.69	0.75	0.00	0.61	0.50	0.26	0.68	0.78	0.73	0.64	0.51	0.67	0.83
3-III	0.65	0.52	0.46	0.52	0.45	0.37	0.82	0.61	0.00	0.70	0.68	0.45	0.55	0.49	0.36	0.56	0.32	0.75
4-I	0.38	0.44	0.72	0.79	0.75	0.81	0.75	0.50	0.70	0.00	0.42	0.79	0.87	0.81	0.74	0.46	0.77	0.92
4-II	0.42	0.40	0.68	0.78	0.70	0.77	0.75	0.26	0.68	0.42	0.00	0.65	0.84	0.78	0.71	0.47	0.74	0.86
4-III	0.75	0.60	0.46	0.61	0.52	0.37	0.88	0.68	0.45	0.79	0.65	0.00	0.61	0.59	0.48	0.64	0.38	0.72
5-I	0.84	0.70	0.60	0.36	0.37	0.41	0.93	0.78	0.55	0.87	0.84	0.61	0.00	0.44	0.54	0.73	0.50	0.77
5-II	0.81	0.64	0.62	0.42	0.35	0.47	0.89	0.73	0.49	0.81	0.78	0.59	0.44	0.00	0.59	0.68	0.53	0.75
5-III	0.68	0.56	0.49	0.58	0.52	0.42	0.85	0.64	0.36	0.74	0.71	0.48	0.54	0.59	0.00	0.62	0.35	0.75
6-I	0.37	0.36	0.59	0.68	0.63	0.64	0.76	0.51	0.56	0.46	0.47	0.64	0.73	0.68	0.62	0.00	0.60	0.80
6-II	0.72	0.57	0.41	0.48	0.49	0.30	0.86	0.67	0.32	0.77	0.74	0.38	0.50	0.53	0.35	0.60	0.00	0.72
6-III	0.92	0.78	0.76	0.76	0.74	0.72	0.95	0.83	0.75	0.92	0.86	0.72	0.77	0.75	0.75	0.80	0.72	0.00
Mean	0.62	0.52	0.55	0.58	0.54	0.53	0.77	0.59	0.51	0.65	0.61	0.57	0.60	0.59	0.55	0.56	0.52	0.75

1–6 – individual foals; I – first sampling time point (within 12 h of birth); II – second sampling time point (after the first month of life); III – third sampling time point (after the second month of life)
